# A rare case of interventricular hydatid cyst in a 17-year-old male: A case report

**DOI:** 10.1016/j.amsu.2022.104058

**Published:** 2022-06-25

**Authors:** Mohamad Abbas, Mohammad Malek Shaghaleh, Mohammad Noor Sultan, Amani Aljress, Mohamad Fateh Kashkash

**Affiliations:** a Pulmonology Department, Aleppo University Hospital, Aleppo, Syria; b Cardiology Department, Aleppo University Hospital, Aleppo, Syria; c Faculty of Medicine, University of Aleppo, Aleppo, Syria

**Keywords:** Hydatid cyst, Echinococcosis, Ventricular septum, Case report, CXR, chest x-ray, ECG, electrocardiogram, CT, computed tomography, WBC, white blood cells, CRP, c-reactive protein, MRI, magnetic resonance imaging

## Abstract

**Introduction and importance:**

Echinococcus infection affects the liver and lungs. However, in unusual cases it may take location in the heart. Furthermore, the settling of the cysts in the interventricular septum are especially rare.

**Case presentation:**

We report a case of a 17-years old male presented with dyspnea and productive cough. Ultrasound showed an incidental finding of a cyst in the distal part of the interventricular septum and the apex of left ventricle. He was treated with a course of antibiotics with albendazole 800 mg then was referred to the cardiac surgery department. The cyst with all its layers was resected.

**Clinical discussion:**

Cardiac hydatid cyst is extremely rare. Especially the involvement of the interventricular septum as it is responsible for only 4% of cardiac cases. Its symptoms maybe nonspecific and the diagnoses is based on echography and computed tomography. The surgical treatment under cardiopulmonary by-bass with complementary course of albendazole 800 mg seems to have a good prognostic outcome.

**Conclusion:**

Cardiac echinococcus should be kept in mind in endemic regions. The diagnosis should be made in the early and uncomplicated stages since it may be fatal. Echocardiography is sensitive and useful for the diagnosis.

## Highlights

• Incidence of hydatid cysts in the interventricular septum is extremely rare.
• Echocardiography and computed tomography presents excellent diagnostic procedures.
• Cardiac hydatid disease should be diagnosed in early stages as it may be fatal.
• Surgical excision and long therapy with albendazole is the preferred treatment.

## 1 Background

Cystic echinococcosis (CE) also known as Hydatid Cystic disease, is caused by the infection with Echinococcus granulosus in its larval stage. It is a parasitic disease that affects humans in endemic regions of the world. In general, this infection takes place in the liver and lungs more than other organs. Only 0.5%–2% of the infected patients have heart involvement, they commonly have previous diagnosis of hydatid cystic disease, only 4% of cardiac cases experience primary involvement of interventricular septum [1].

Cardiac hydatidosis can be asymptomatic, in some cases the patient could complain of chest pain, dyspnea, palpitations, and cough as the primary symptoms [2]. The diagnosis is challenging and usually it is masquerade with other pulmonary diseases.

In this case, we are presenting an unusual case of a hydatid cyst located between the distal part of the interventricular septum and left ventricular apex, in a 17-year-old male with nonspecific symptoms and a past medical history of tuberculosis (TB) infection.

This work has been reported in line with the SCARE criteria [3].

## 2 Objective

To report a rare case of cardiac hydatid disease in the distal part of the interventricular septum and apex of left ventricle.

## 3 Case report

A 17-year-old adolescent presented to the emergency department. He was complaining of severe dyspnea, cough, palpitation and fever. He had a history of pulmonary tuberculosis which was treated with the full therapy one year before presentation. The patient had no other family or surgical history.

The physical examination revealed cyanosis and the using of accessory muscles. SpO2 was 70% with room air which improved to 92% with high flow oxygen. Respiratory rate was 35\min and the heart rate was 120\min. There was no erythema or signs of lymphadenopathy.

**Fig. 1:**
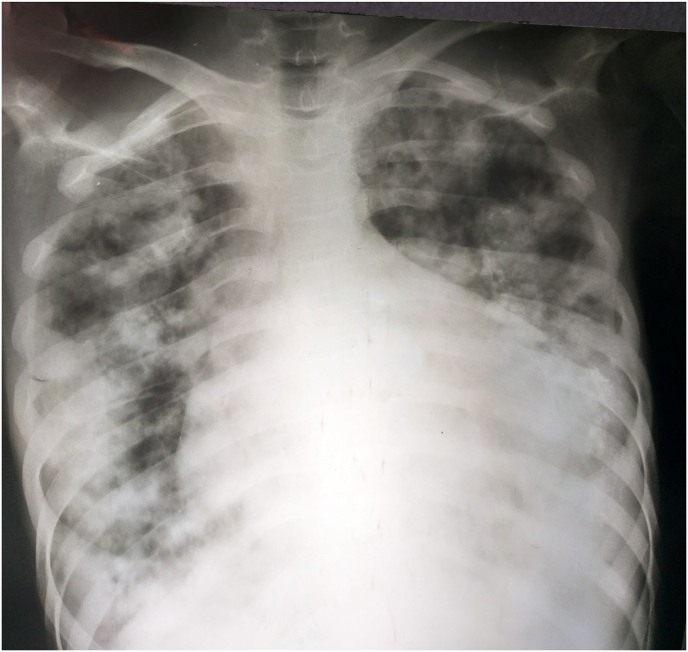
Chest X ray shows multiple well-defined opacities with cavities in some of them and increased cardiothoracic-ratio.

Laboratory tests showed total leukocyte count of 15,9 × 10^9^ \L, hemoglobin of 10.1 g\dl, CRP of 80 g\dl and normal eosinophils count. Serology test results for hydatid cysts (ELISA) was positive. CXR showed increased cardiothoracic-ratio and multiple well-defined opacities with cavities in some of them (Fig. 1). The most important differential diagnoses that had to be excluded is reactivation of a latent tuberculosis, therefore tuberculosis tests were also taken. There were no acid-fast bacilli in three sputum samples and no mycobacterium tuberculosis complex (MTBC) were detected using cepheid's xpert MTB\RIF assay. ECG showed poor R wave progression, low voltage in the anterior leads (V1–V6).

**Fig. 2:**
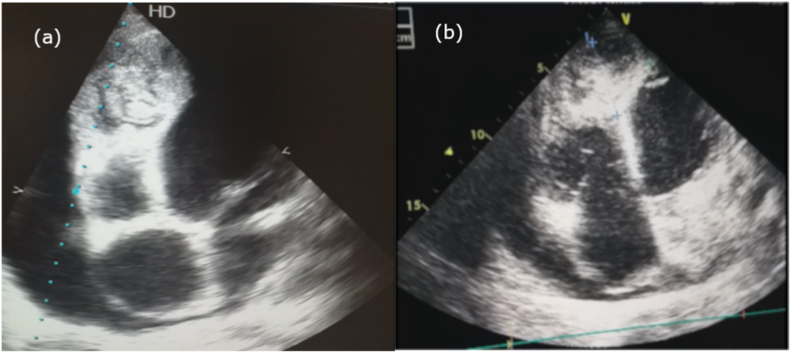
Transthoracic echocardiography shows (a) a hydatid cyst in the distal part of the interventricular septum and apex of left ventricle (b) dimensions of the cyst (6 × 4.3 cm).

**Fig. 3:**
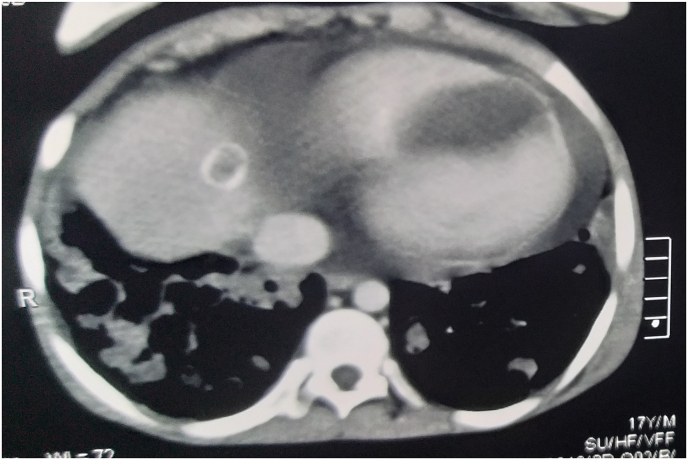
Transverse computed tomography shows hydatid cyst in the distal part of the interventricular septum.

The patient was referred to the cardiology unit where he underwent an echocardiography that showed a 6 × 4.3 cm cystic formation in distal part of the interventricular septum and apex of left ventricle (Fig. 2). Thoracic multislice computed tomography (CT) showed multiple cystic lesions with air–fluid levels in both lungs in addition to the cystic formation in the interventricular septum (Fig. 3). In abdominal CT scan, two small hypo dense lesions were seen in the liver. Depending on the physical findings and the radiological manifestations a diagnosis of a hydatid cyst was made.

Finally, the patient was treated with a course of antibiotics with albendazole 800 mg in addition to intravenous paracetamol 1000mg to relief pain. After seven days, he has improved clinically with significant decrease in values of WBC and CRP. Then he was referred to the cardiac surgery department and underwent surgery to resect the cyst with all its layers under cardiopulmonary by-bass. The surgical treatment was performed by a senior cardiac surgeon aided by a junior resident. Patient received complementary course of albendazole 800 mg to reduce risk if recurrence.

Postoperatively, the patient was followed up for two weeks with improvement in symptoms and no recurrence manifestations.

## 4 Discussion and conclusion

Hydatid cystic disease is an endemic disease in Syria and other livestock-raising countries [3]. The most common sites of hydatid cysts are the liver (in 50%–70% of cases), lungs (5%–30%), muscles (5%), bones (3%), kidneys (2%), spleen (1%), and brain (1%) [4].

Here we report an unusual case of a hydatid cyst between the distal section of the interventricular septum and the left ventricular apex. Cardiac hydatid cyst disease is a rare (0.5%–2%), but potentially fatal pathology [5]. It may mimic valvular lesions, give signs of an intracardiac mass, or lead to a congestive heart failure [5]. Cardiac involvement usually occurs during adulthood and maybe isolated or associated with cysts in other organs, especially lungs and liver as in our patient [5].

As long as, the latent phase between infection and presentation of the disease is long and the symptoms may be nonspecific, establishing an early diagnosis is difficult [5]. The coronary circulation is the main pathway by which the parasitic larvae reach the heart. The left ventricle is the site of cardiac hydatid cysts in 55%–60% of cases because of the rich coronary blood supply. Less frequently, we can see it in the right ventricle (10%–15% of cases), pericardium (7%), pulmonary artery (6%–7%), left atrium (6%–8%), right atrium (3%–4%), and interventricular septum (4%). Although cough is typically the chief clinical symptom of hydatid disease, cardiac hydatid cysts are usually asymptomatic, especially in their early stages, only 10% of patients have clinical symptoms. Chest pain is the most common symptom; however, palpitations, cough and dyspnea are also seen, as in our patient [4].

Chest radiographs usually show a normal cardiothoracic-ratio but it is sometimes large with signs of lung hydatid cysts, as in our patient [4].

Electrocardiographic findings vary according to the sites of the cysts and are usually not specific. Echocardiography is sensitive for diagnosis of cardiac hydatid cyst as we found in our case. However, it is necessary to do CT scan or MRI to find additional information about the accurate location of the lesion in addition to the relation of it with other structures [6].

Serologic tests can be false-negative in 10%–20% of patients in hepatic hydatid cysts, 40% in pulmonary cysts, and 50% in cardiac cysts; this is most likely linked with an insufficient immune response [4]. However, the ELISA is one of the most specific serologic tests that can be used, and a positive result for echinococcus antibodies confirms the diagnosis [4].

Surgical excision and long-term therapy with albendazole is the preferred treatment [4].

## 5 Conclusion

Cardiac hydatid disease although rare, remains a significate medical condition and should always be in the differential diagnoses and excluded in the initial workup with any patient presenting with cardiac or respiratory symptoms in areas where the disease is endemic. Echocardiography alongside CT scan present excellent procedures to precisely diagnose the cysts, measure their diameters and determine their relation to surrounding structures.

## Sources of funding

None.

## Ethical approval

The study adhered the tenets of the Declaration of Helsinki.

## Consent

Written informed consent was obtained from the patient for publication of this case report and accompanying images. A copy of the written consent is available for review by the Editor-in-Chief of this journal on request.

## Author contribution

**MA and MS** made therapeutic regimen and followed up the patient, obtained patient's consent, wrote in the Original Draft, reviewed the literature, reviewed & edited the manuscript, **MA** and **MS** contributed equally to this paper. **AA** wrote up part of the original draft, reviewed the literature, and reviewed the final manuscript. **MS** wrote up part of the original draft, prepared the figures, and reviewed & edited the final manuscript. **MK** examined and followed-up the patient, revised and edited the final manuscript.

All authors have read and approved the manuscript.

## Research registration

N/a.

## Guarantor

Mohamad Abbas.

Email address: mhd-abbas@hotmail.com.

## Provenance and peer review

Not commissioned, externally peer-reviewed.

## Declaration of competing interest

None.
